# Shared Genetic Architecture and Pleiotropic Loci Linking Endometriosis and Inflammatory Bowel Disease: An Integrative GWAS, Colocalization, and Mendelian Randomization Study

**DOI:** 10.1155/bmri/8422273

**Published:** 2026-07-27

**Authors:** Xinyue Cui, Jianhong Wang, Jingjing Chen, Hao Wu, Jiaxuan Liu, Yali Fang, Guiping Wan

**Affiliations:** ^1^ Department of Obstetrics and Gynecology, Affiliated Hospital of Integrated Traditional Chinese and Western Medicine, Nanjing University of Chinese Medicine, Nanjing, China, njucm.edu.cn; ^2^ Department of Obstetrics and Gynecology, Sir Run Run Hospital, Nanjing Medical University, Nanjing, China, njmu.edu.cn; ^3^ Department of Obstetrics and Gynecology, Nanjing Tongren Hospital, Nanjing, China

**Keywords:** endometriosis, genetic pleiotropy, GWAS, inflammatory bowel disease, Mendelian randomization

## Abstract

**Background:**

Endometriosis (EMS) and inflammatory bowel disease (IBD) are both chronic inflammatory disorders with overlapping clinical features, suggesting a potentially shared etiology. Although epidemiological studies have reported an association between the two conditions, the genetic basis of this relationship remains poorly understood. This study is aimed at characterizing the shared genetic architecture of EMS and IBD, identify pleiotropic loci implicated in both conditions, and explore the potential causal relationships and underlying biological mechanisms.

**Methods:**

We analyzed large‐scale GWAS summary statistics to investigate the genetic relationship between EMS and IBD. Linkage‐disequilibrium score regression (LDSC) was used to estimate genetic correlation. The PLACO method was applied to identify pleiotropic loci, which were further evaluated by Bayesian colocalization. Functional implications were assessed using MAGMA gene‐set analysis, tissue enrichment analysis, and functional annotation. Bidirectional two‐sample Mendelian randomization (MR) was performed to examine causal inference, accompanied by leave‐one‐out sensitivity analyses and statistical power calculations.

**Results:**

A statistically significant but modest positive genetic correlation was observed between EMS and IBD (r_g_ = 0.0957, *p* = 0.0042). Twenty‐four genomic loci showed evidence of pleiotropic effects, and six of these carried strong evidence of a shared causal variant (posterior probability: PP.H4 > 0.7), including loci at 2p23.3 (ADCY3/DNAJC27), 4q12, 5q23.3, 7p15.2, 11q13.1 (CCDC88B), and 1q22. Functional analyses consistently implicated eight pleiotropic genes across multiple platforms: ADCY3, CCDC88B, GREB1, NOD2, RSPO3, SYNE1, THADA, and TRAIP. Pathway enrichment highlighted “positive regulation of gene expression” as a key shared mechanism (*p* < 0.05). Tissue‐specific analyses revealed strong signals in whole blood, spleen, and colon, consistent with the immunoinflammatory etiology of both diseases. Bidirectional MR did not support a causal relationship in either direction (IBD on EMS: OR = 1.007, 95% CI: 0.986–1.028, *p* = 0.543; EMS on IBD: OR = 1.054, 95% CI: 0.991–1.122, *p* = 0.092). Leave‐one‐out analyses confirmed that no single variant drove these estimates, and power analysis indicated adequate power to detect moderate causal effects.

**Conclusions:**

The findings provide evidence for a shared, albeit modest, and genetic basis between EMS and IBD that is likely mediated through immune regulation, hormonal signaling, and tissue repair. The absence of significant causal effects in MR suggests that the observed comorbidity reflects common genetic susceptibility rather than a direct causal link. These results offer new insight into the mechanisms underlying the co‐occurrence of the two conditions and may inform future investigation of shared therapeutic targets.

## 1. Introduction

Endometriosis is a common gynecological disorder, defined by the presence of endometrial‐like tissue outside the uterus, that affects approximately one in 10 women of reproductive age [1]. Patients typically present with chronic pelvic pain, dysmenorrhea, and infertility, and the disease is increasingly viewed as a systemic inflammatory condition rather than a purely local pelvic disorder. Inflammatory bowel disease (IBD), which comprises Crohn′s disease and ulcerative colitis, is an immune‐mediated gastrointestinal disorder whose global incidence continues to rise [2]. The two diseases share overlapping symptoms: Women with EMS frequently report gastrointestinal discomfort, and female patients with IBD may experience menstrual irregularities [3]. Epidemiological data indicate that women with EMS have an approximately 50% higher risk of developing IBD than controls [4]. However, because observational studies are susceptible to confounding and reverse causation, genetic approaches are needed to clarify the nature of this association.

A growing body of evidence suggests that endometriosis shares biological features with systemic autoimmune and inflammatory diseases, with the immune–hormonal axis playing a central role in both EMS and IBD. In endometriosis, the peritoneal environment is characterized by elevated levels of proinflammatory cytokines, including tumor necrosis factor‐*α* (TNF‐*α*), interleukin‐6 (IL‐6), and interleukin‐1*β* (IL‐1*β*), together with dysregulated activity of natural killer cells and macrophages [5]. IBD is similarly driven by aberrant mucosal immune responses that involve the same cytokines and immune‐cell populations [6]. Both conditions involve activation of the NF‐*κ*B signaling pathway, a master regulator of inflammatory gene expression [7]. Moreover, estrogen modulates intestinal immune homeostasis, and fluctuations in estrogen levels can influence the course of IBD in female patients [8], pointing to a hormonal link between the two diseases.

At the genetic level, IBD has been shown to share susceptibility loci with a range of autoimmune disorders, including rheumatoid arthritis, ankylosing spondylitis, and psoriasis [9]. However, the extent of genetic overlap between IBD and gynecological conditions such as endometriosis has not been systematically investigated. Although GWAS have identified numerous susceptibility loci for EMS and IBD individually, few studies have integrated these datasets to uncover shared genetic architecture. Methods such as cross‐trait linkage‐disequilibrium score regression (LDSC), pleiotropic analysis under the composite null hypothesis (PLACO), and Bayesian colocalization now enable the systematic identification of loci that confer risk for multiple traits simultaneously.

To address this gap, we applied an integrative genomic approach that combined LDSC genetic correlation analysis, PLACO pleiotropy detection, Bayesian colocalization, gene‐set and tissue enrichment analyses, and bidirectional two‐sample Mendelian randomization. By identifying pleiotropic loci together with the genes and pathways they implicate, this study is aimed at elucidating the molecular mechanisms that may underlie the epidemiological co‐occurrence of endometriosis and IBD.

## 2. Materials and Methods

### 2.1. Study Design and Data Sources

To evaluate the genetic correlation between endometriosis and IBD, we used LDSC as the primary analytical approach. Pleiotropic loci were identified with the PLACO method, and causal relationships were examined through bidirectional two‐sample MR analysis. IBD GWAS data were obtained from a large meta‐analysis [10] that included 25,305 subjects and, through integration with publicly available summary statistics, reached a combined sample size of 59,957 individuals. The analysis identified 25 novel susceptibility loci, several of which encode therapeutic integrin targets. Endometriosis GWAS data were obtained from the FinnGen R12 release (https://r12.finngen.fi/, 2023) and comprised 20,190 cases and 130,160 controls. The Finnish Biobank dataset (approximately 500,000 participants) was used, with sex, age, 10 ancestry principal components, and genotyping batch included as covariates to control for confounding. Exposure and outcome samples were not overlapping, which avoided sample‐overlap bias. Each original GWAS was approved by its respective institutional review board; the present study used only publicly available summary‐level statistics and therefore did not require additional ethical approval.

### 2.2. Genetic Correlation Analysis

Genome‐wide shared heritability was quantified using LDSC [11] (v1.0.1, PMID: 26414676). LD scores were computed from the 1000 Genomes Phase 3 European panel and HapMap3 reference sample [12]. Variants were excluded if they were nondiploid, strand‐ambiguous, duplicated, or lacking an rsID, had a minor − allele frequency ≤ 0.01, or mapped to the major histocompatibility complex region (chr6: 28.5–33.5 Mb, hg19). Genetic correlation (r_g_) and its standard error were estimated using two‐tailed tests, with significance defined as *p* < 0.05.

### 2.3. Pleiotropy Analysis Under the Composite Null Hypothesis

SNP‐level PLACO integrates summary association statistics to identify pleiotropic loci across traits [13]. Squared Z statistics (*Z* ^2^) were computed for each variant, and extreme values (*Z* ^2^ > 80) were truncated to mitigate the influence of outlier variants with disproportionately large effects. The *Z*‐score covariance matrix was obtained by Spearman correlation, and the intersection–union test was used to evaluate the null hypothesis of no pleiotropy. Bonferroni correction, based on the total number of tested SNPs, was applied to control the family‐wise error rate. For multiallelic regions annotated in Functional Mapping and Annotation of Genetic Associations (FUMA), Bayesian colocalization analysis was performed with the R package coloc (v5.1.0, PMID: 27866706) [14] to identify putative common causal variants at each locus. Under the assumption of a single causal variant, colocalization tests five scenarios: no genetic association in the region (Hypothesis 0), association only with IBD (Hypothesis 1), association only with EMS (Hypothesis 2), independent causal variants (Hypothesis 3), or a single shared causal variant for both traits (Hypothesis 4). The analysis used coloc. abf with parameters p1 = p2 = 1 × 10^−4^ and p12 = 1 × 10^−5^; a posterior probability of PP.H4 > 0.7 was considered strong evidence of shared causality.

### 2.4. Functional Analysis of Pleiotropic Loci

On the basis of the PLACO results, the identified pleiotropic loci were mapped to nearby genes to explore their shared biological mechanisms. For genes located at or overlapping with pleiotropic loci based on PLACO output and univariate GWAS, MAGMA was used to perform gene‐set analysis [15] and to characterize the tissue‐specific enrichment of pleiotropic candidate pathways and genes. The FUMA platform was used to interpret the biological functions of the pleiotropic loci [16]. A series of pathway enrichment analyses was conducted against the Molecular Signatures Database (MSigDB) to further investigate the functions of the mapped genes [17]. In addition, eQTL analysis incorporated SNP–gene association data from multiple tissues, including whole blood.

### 2.5. Mendelian Randomization Analysis

Instrumental variables were selected from the full GWAS summary statistics of each exposure using the PLINK (v1.9) clumping algorithm [18]. For the primary (forward) analysis (IBD on EMS), the conventional genome‐wide significance threshold was used (*p* < 5 × 10^−8^). For the reverse analysis (EMS on IBD), because no SNP reached genome‐wide significance (*p* < 5 × 10^−8^) in the endometriosis GWAS, a relaxed threshold of *p* < 5 × 10^−6^ was adopted, a strategy widely used in MR studies of traits with underpowered GWAS or limited genetic instruments [19]. LD clumping was performed with *r* ^2^ = 0.001 and a 10,000‐kb window in the 1000 Genomes Phase 3 European reference panel. SNPs associated with known confounders such as BMI or smoking (*p* < 0.01) were excluded on the basis of PhenoScanner queries.

Instrument strength was assessed with the per‐variant *F*‐statistic, calculated as *F* = (*β*/SE)^2^, where *β* and SE are the effect size and its standard error from the exposure GWAS [19]; an *F*‐statistic greater than 10 was considered indicative of strong instrument strength. This pipeline yielded 109 independent SNPs for the forward analysis (mean *F* = 66.5, minimum *F* = 29.8) and 21 independent SNPs for the reverse analysis (mean *F* = 64.3, minimum *F* = 30.2); all instruments exceeded the *F* > 10 threshold.

The inverse variance‐weighted (IVW) method served as the primary estimator. Complementary sensitivity analyses included MR‐Egger regression, weighted median, weighted mode, debiased inverse variance‐weighted (DIVW), and MR‐RAPS. Between‐instrument heterogeneity was assessed with Cochran′s *Q* test for both the IVW and MR‐Egger models [20]. The MR‐Egger intercept was used to test for directional horizontal pleiotropy [21]. To determine whether the overall estimates were driven by any single instrument, leave‐one‐out (LOO) sensitivity analyses were performed by sequentially excluding each SNP and recomputing the IVW estimate. Forest plots summarizing the LOO results are provided in Figures S8 and S9, with per‐SNP results in Table S10.

Statistical power was estimated using the noncentral chi‐square framework of Brion et al. [22] as implemented in the mRnd tool (https://shiny.cnsgenomics.com/mRnd/), based on the observed proportion of exposure variance explained by the instruments (*R* ^2^), the outcome case/control counts, and a two‐sided *α* of 0.05. Complete power calculations across a range of detectable odds ratios are reported in Table S11.

### 2.6. Statistical Analysis

All analyses were performed in R (v3.5.3). MR analyses were conducted with the MendelianRandomization R package (v0.5.0) [23]. A nominal *p* < 0.05 was considered statistically significant. Multiple‐testing correction was applied using either Bonferroni or Benjamini–Hochberg false‐discovery rate procedures as appropriate. Visualizations (Manhattan, regional, and enrichment plots) were produced with ggplot2 (v3.3.5). Complete analysis scripts and parameter files are provided as a reproducibility package (see Data Availability Statement).

## 3. Results

### 3.1. Identification of Pleiotropic Loci

**Figure 1:**
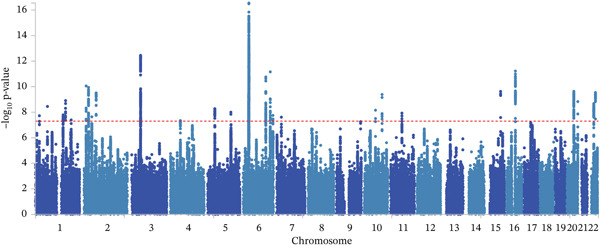
Manhattan plot of pleiotropic loci between IBD and EMS.

To investigate the shared genetic basis of EMS and IBD, we performed cross‐trait analysis with PLACO and identified 24 genomic loci with potential pleiotropic effects between the two diseases (Figure 1; the genomic size, number of candidate SNPs, and number of mapped genes for each locus are summarized in Figure S2). The Manhattan plot (Figure 1) highlights significant signals across multiple chromosomes, with *p* values ranging from 3.54 × 10^−13^ (3p21.31) to 4.51 × 10^−8^ (4q12). These findings were further supported by coloc‐based colocalization analysis, which quantified the posterior probability (PP.H4) for a shared causal variant at each locus. Six loci showed strong evidence of pleiotropy (PP.H4 > 0.7): 2p23.3 (rs2384054, rs56197924; PP.H4 = 0.814), 4q12 (rs13143894; PP.H4 = 0.799), 5q23.3 (rs2107607; PP.H4 = 0.878), 7p15.2 (rs3757639; PP.H4 = 0.892), 11q13.1 (rs479552; PP.H4 = 0.938), and 1q22 (rs2948071; PP.H4 = 0.764) (Table 1). Regional plots of these loci (Figure 2A–F: 1q22, 2p23.3, 4q12, 5q23.3, 7p15.2, and 11q13.1) show concentrated signal distributions, supporting shared genetic effects at each region. The QQ plot (Figure S1) did not display genomic inflation, confirming the stability of the pleiotropy signals.

**Table 1:** Characteristics of the pleiotropic loci identified.

Genomic locus	Locus boundary	Lead SNPs	*p* value	Mapped genes	PP H4
1p36.12	1:22248466‐22586280	rs2501281	1.89E‐08	LINC00339, CDC42	0.158
1p31.3	1:67597693‐67764815	rs10489628	3.56E‐09	IL23R	0.158
1q22	1:154988957‐156079630	rs2948071	1.62E‐08	GON4L	0.764
1q32.1	1:200473479‐201070422	rs59682551	4.05E‐08	KIF21B	0.094
2p25.1	2:11605381‐11754965	rs17529680	8.78E‐11	GREB1	0.082
2p23.3	2:24657179‐25625422	s2384054; rs56197924	1.13E‐10	ADCY3, DNAJC27	0.814
2p21	2:43449385‐43912735	rs13399366	2.30E‐08	THADA	0.564
2p14	2:67446530‐67972623	rs6546324	3.09E‐10	AC010987.5	0.149
3p21.31	3:48866313‐50521402	rs10865958	3.54E‐13	AC139451.1, TRAIP	0.360
4q12	4:55883795‐55931327	rs13143894	4.51E‐08	RP11‐530I17.1	0.799
5p13.1	5:39518437‐40533309	rs1395158	5.30E‐09	GCSHP1, LINC00603	0.681
5q23.3	5:129448270‐131374882	rs2107607	1.00E‐08	RP11‐114H7.3, HINT1	0.878
6q22.33	6:126614101‐127849425	rs9482772	1.73E‐11	RSPO3	0.451
6q25.2	6:152538154‐153272408	rs7741405; rs9479275; rs9479384	6.90E‐12	SYNE1	0.175
6q27	6:167346133‐167547442	rs6941355	1.78E‐08	RP11‐517H2.6	0.096
7p15.2	7:26668837‐27312951	rs3757639	2.46E‐08	HOXA‐AS3, RP1‐170O19.22	0.892
10q21.2	10:64266932‐64699179	rs10160102	6.98E‐09	ZNF365	0.077
10q24.2	10:101231799‐101350225	rs4590800	4.08E‐10	NKX2‐3, snoU13	0.177
11q13.1	11:63810915‐64218143	rs479552	1.18E‐08	CCDC88B	0.938
16q12.1	16:50659759‐50846162	rs3135499	5.96E‐12	NOD2	0.288
20q12	20:39577519‐40270138	rs17181845	2.31E‐10	ZHX3	0.676
20q13.33	20:62191558‐62770956	rs310669	1.48E‐09	HELZ2, GMEB2	0.533
22q12.2	22:29860811‐30714957	rs62226366	2.11E‐09	CTA‐85E5.10	0.630
22q13.1	22:39607926‐39972069	rs75026781	2.86E‐10	RPL3, SYNGR1	0.317

Figure 2Regional plots of pleiotropic loci (Panels (A–F): 1q22, 2p23.3, 4q12, 5q23.3, 7p15.2, and 11q13.1).

**Figure:**
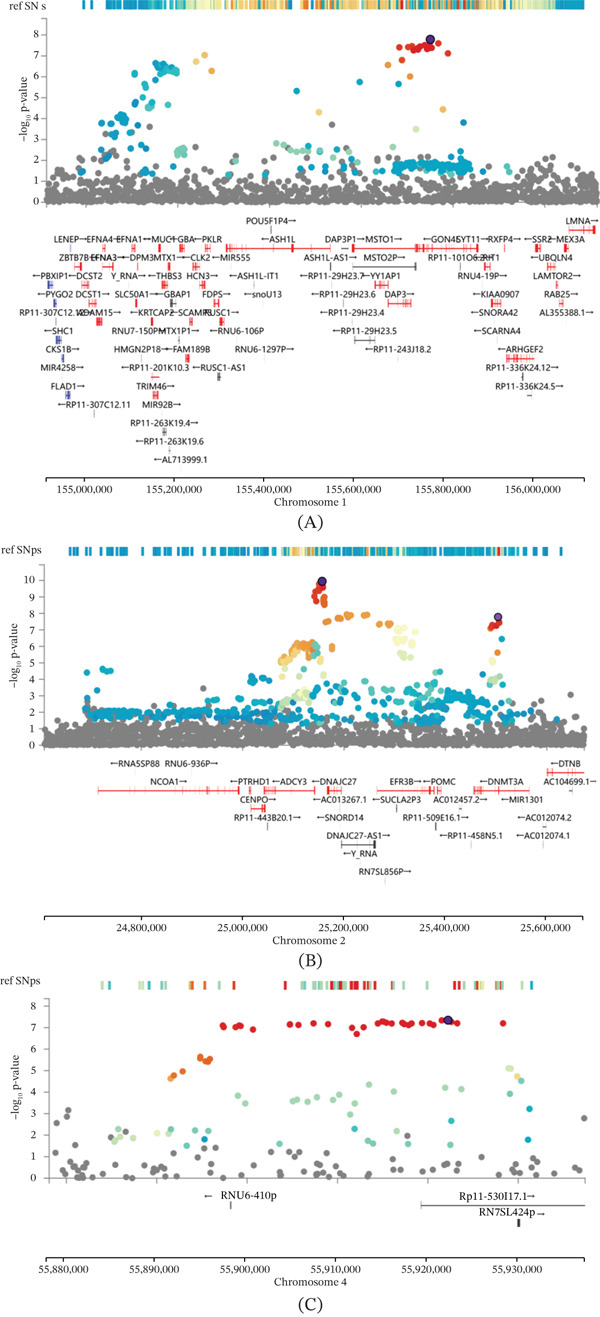
(a)

**Figure:**
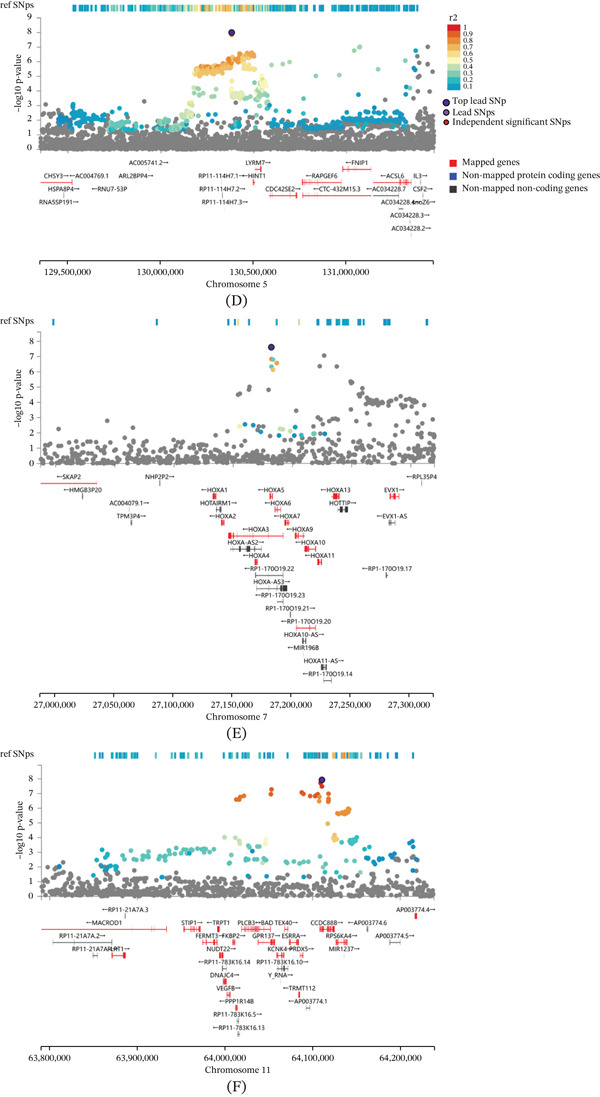
(b)

### 3.2. Genetic Correlation Between Endometriosis and IBD

LDSC analysis revealed a statistically significant positive genetic correlation between IBD and EMS (r_g_ = 0.0957, *p* = 0.0042). Although statistically significant, this correlation was modest in magnitude, indicating limited rather than broad polygenic overlap between the two conditions. This finding provided a rationale for targeted analyses of specific pleiotropic loci and their functional implications. Quality‐control measures included exclusion of nondiploid SNPs, strand‐ambiguous SNPs, SNPs with missing rsIDs, duplicated SNPs, SNPs with MAF < 0.01, and SNPs within the MHC region (chr6: 28.5–33.5 Mb), ensuring the robustness of the LDSC estimates.

### 3.3. Functional Annotation and Gene Mapping

**Figure 3:**
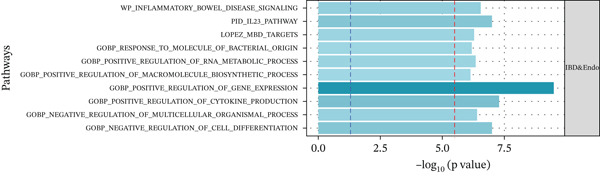
MAGMA gene‐set enrichment analysis based on genome‐wide pleiotropy.

**Figure 4:**
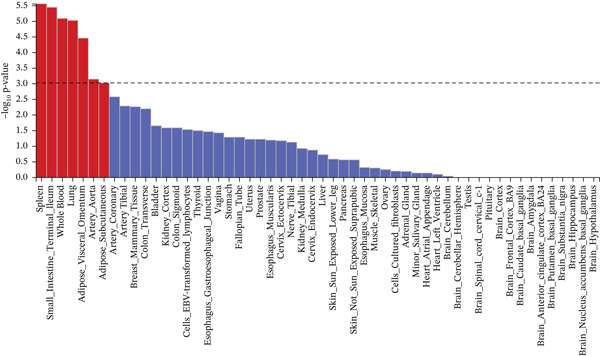
Tissue‐specific expression analysis of genome‐wide pleiotropic loci based on MAGMA (54 GTEx tissues).

Functional mapping of the 24 pleiotropic loci identified 33 neighboring genes based on lead‐SNP position (Table 1), with detailed annotations for these genes—including Ensembl and Entrez identifiers, OMIM entries, and druggability information—provided in Table S5. MAGMA gene‐set analysis, applied to the full distribution of SNP *p* values, identified 45 pleiotropic genes associated with these loci (Figure 3); the corresponding Manhattan and quantile–quantile plots of the gene‐level analysis are shown in Figures S4 and S5, and the complete gene‐level results are listed in Table S3. eQTL analysis (GTEx V8, 54 tissues) linked 355 genes to the risk variants (Figure 4), with the full list of eQTL‐mapped genes by tissue provided in Table S4. Notably, eight genes—ADCY3, CCDC88B, GREB1, NOD2, RSPO3, SYNE1, THADA, and TRAIP—were detected by all three methods, providing convergent evidence for their pleiotropic involvement. Assessment of the functional impact of the pleiotropic SNPs (Figure S3) showed that a substantial proportion were located in intronic regions, suggesting regulatory roles. The regional plots (Figure 2) further illustrated the positional relationships between lead SNPs and mapped genes, with particularly strong associations at ADCY3 and DNAJC27 (2p23.3) and at CCDC88B (11q13.1).

### 3.4. Pathway and Tissue Enrichment Analysis

**Figure 5:**
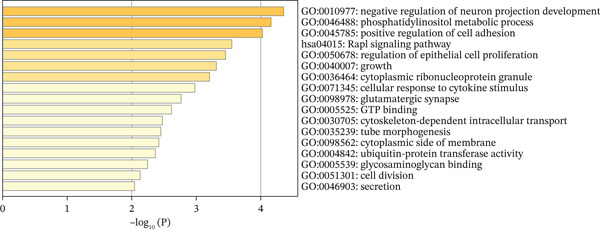
Enriched pathways associated with pleiotropic genes.

MAGMA gene‐set enrichment analysis identified 10 significantly enriched pathways, of which positive regulation of gene expression was the most prominent (*p* < 0.05 after correction; Figure 3), with the complete gene‐set enrichment statistics reported in Table S2. This pathway involves genes such as ADCY3 and NOD2 and highlights a potential role for transcriptional regulation in the shared biology of IBD and EMS. Tissue‐specific enrichment analyses across 54 GTEx tissues revealed significant signals in whole blood, spleen, and colon (Figure 4). Enrichment in whole blood (*p* < 0.01) reflected systemic immune involvement, whereas enrichment in the spleen (*p* < 0.05) and colon (*p* < 0.01) aligned with the inflammatory pathology of IBD and the tissue‐specific manifestations of EMS. eQTL analysis showed elevated expression of ADCY3, CCDC88B, and NOD2 in whole blood and EBV‐transformed lymphocytes (Figure S6), and the complete tissue‐by‐gene expression matrix across the 54 GTEx tissues is provided in Table S6. Pathway enrichment of the pleiotropic genes (Figure 5; the corresponding GO and KEGG enrichment results are listed in Table S7) and the protein–protein interaction (PPI) network (Figure S7) further underscored the interconnectedness of these genes within immune signaling cascades.

### 3.5. Mendelian Randomization Analysis

Causal inference was performed with a bidirectional two‐sample MR framework. After PLINK LD clumping (*p* < 5 × 10^−8^ forward; *p* < 5 × 10^−6^ reverse; *r* ^2^ = 0.001; 10,000‐kb window; 1000 Genomes Phase 3 EUR reference), 109 independent SNPs were retained as instruments for the forward analysis (IBD on EMS) and 21 for the reverse analysis (EMS on IBD). All instruments had *F*‐statistics greater than 10 (forward: mean *F* = 66.5, minimum *F* = 29.8; reverse: mean *F* = 64.3, minimum *F* = 30.2), confirming adequate instrument strength. The cumulative proportion of exposure variance explained by the instruments was 12.06% for the forward analysis and 0.90% for the reverse analysis.

**Figure 6:**
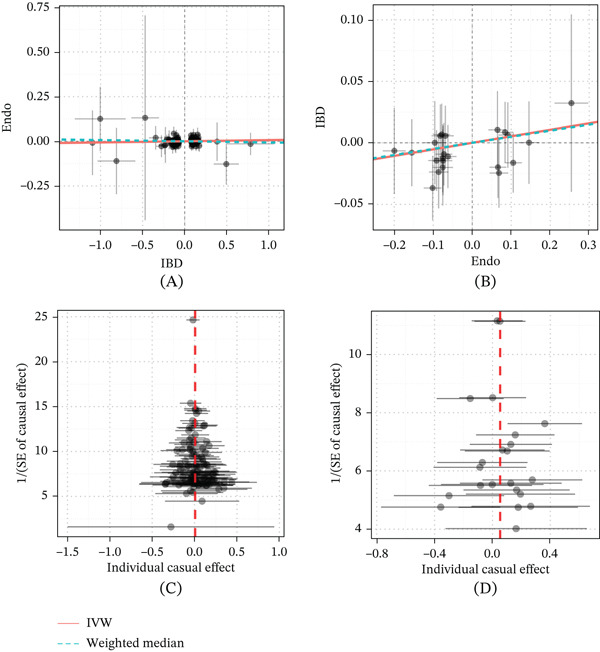
Bidirectional Mendelian randomization analysis between IBD and EMS. (A) Scatter plot of the causal effect of IBD on endometriosis. (B) Scatter plot of the causal effect of endometriosis on IBD. (C) Funnel plot of the causal effect of IBD on endometriosis. (D) Funnel plot of the causal effect of endometriosis on IBD.

In the primary IVW analysis, the OR for IBD on EMS was 1.007 (95% CI: 0.986–1.028, *p* = 0.543), and the OR for EMS on IBD was 1.054 (95% CI: 0.991–1.122, *p* = 0.092); neither direction reached statistical significance (Table 2). These findings were consistent across all complementary methods, including MR‐Egger, weighted median, weighted mode, DIVW, and MR‐RAPS (Table 2). MR‐Egger intercept tests did not indicate directional pleiotropy (*p* = 0.171 for IBD on EMS; *p* = 0.799 for EMS on IBD), and Cochran′s *Q* tests did not reveal significant heterogeneity among the instrument‐specific estimates (*Q* = 22.632, *p* = 0.205 for IBD on EMS; *Q* = 23.202, *p* = 0.333 for EMS on IBD). LOO analyses confirmed that no single SNP disproportionately influenced the causal estimates: Across the 109 forward LOO iterations, the causal OR ranged from 0.986 to 0.992 (all *p* > 0.05), and across the 21 reverse iterations the OR ranged from 1.035 to 1.060 (all *p* > 0.05). Per‐SNP LOO results are reported in Table S10, with forest plots in Figures S8 and S9. Scatter plots (Figure 6A,B) and funnel plots (Figure 6C,D) also supported the absence of a causal association.

**Table 2:** MR analysis results.

	Methods	Estimate	*p*	Heterogeneity test	
				Estimate	*p*
IBD on Endo	IVW (fixed)	1.007 (0.986, 1.028)	0.543	22.632	0.205
	IVW (random)	1.007 (0.984, 1.029)	0.565		
	MR‐Egger (slope)	0.972 (0.92, 1.027)	0.309		
	MR‐Egger (intercept)	0.005 (−0.002, 0.011)	0.171		
	Weighted mode	0.986 (0.935, 1.04)	0.597		
	Weighted median	0.994 (0.962, 1.026)	0.694		
	DIVW	1.007 (0.984, 1.03)	0.563		
	MR‐RAPS	1.007 (0.985, 1.028)	0.543		
Endo on IBD	IVW (fixed)	1.054 (0.991, 1.122)	0.092	23.202	0.333
	IVW (random)	1.054 (0.988, 1.125)	0.109		
	MR‐Egger (slope)	1.081 (0.876, 1.333)	0.451		
	MR‐Egger (intercept)	−0.002 (−0.023, 0.018)	0.799		
	Weighted mode	1.057 (0.949, 1.177)	0.315		
	Weighted median	1.051 (0.96, 1.15)	0.281		
	DIVW	1.055 (0.99, 1.125)	0.101		
	MR‐RAPS	1.055 (0.991, 1.124)	0.092		

Power analysis based on the observed *R* ^2^ indicated that the forward MR had 88.7% power to detect an OR of 1.05 and > 99.9% power for any OR ≥ 1.10 at *α* = 0.05. The reverse analysis was more modestly powered, owing to the smaller exposure variance explained and the smaller outcome sample size: power was 34% for OR = 1.10, 84% for OR = 1.20, 99% for OR = 1.30, and > 99.9% for any OR ≥ 1.50. These estimates indicate that the null finding in the forward direction is very unlikely to reflect insufficient statistical power, whereas the reverse direction has adequate power only for moderate‐to‐large effects. Complete power calculations are presented in Table S11, and the corresponding power curves are shown in Figure S10.

## 4. Discussion

This study systematically investigated the genetic relationship between EMS and IBD using LDSC, PLACO, and MR. A statistically significant but modest genetic correlation (r_g_ = 0.0957, *p* = 0.0042) was observed between the two conditions, indicating limited but genuine polygenic overlap. PLACO combined with Bayesian colocalization identified 24 pleiotropic loci, of which six (PP.H4 > 0.7) showed strong evidence of shared causal variation, including 1q22 (PP.H4 = 0.764), 2p23.3 (ADCY3/DNAJC27; PP.H4 = 0.814), 4q12 (PP.H4 = 0.799), 5q23.3 (PP.H4 = 0.878), 7p15.2 (PP.H4 = 0.892), and 11q13.1 (CCDC88B; PP.H4 = 0.938). Regional plots (Figure 2) showed that signals at these loci were tightly concentrated, consistent with genetic effects closely coupled within the same genomic regions. However, bidirectional MR did not detect significant causal effects in either direction (*p* = 0.543 for IBD on EMS; *p* = 0.092 for EMS on IBD), suggesting that the observed genetic overlap increases susceptibility to both diseases through common pathways rather than reflecting a unidirectional causal link. This interpretation is supported by LOO analyses, which showed that no single instrument drove the null estimates, and by power analyses, which confirmed adequate power to detect moderate causal effects in the forward direction.

### 4.1. Molecular Mechanisms of the Pleiotropic Loci and Key Genes

Pleiotropic loci were identified with a stringent threshold of PP.H4 > 0.7 in the Bayesian colocalization framework to ensure high‐confidence evidence of shared causal variants. At the 2p23.3 locus, the lead SNPs rs2384054 and rs56197924 mapped to ADCY3 and DNAJC27 (PP.H4 = 0.814), indicating highly overlapping association signals for IBD and EMS. ADCY3 catalyzes the conversion of ATP to cAMP, a second messenger that regulates macrophage polarization through protein kinase A (PKA). cAMP signaling has been reported to promote anti‐inflammatory M2 macrophage polarization through downstream mediators such as IL‐10 and IL‐33, thereby facilitating mucosal repair in IBD [24]. We speculate that impaired ADCY3 function could lead to insufficient local cAMP levels in the intestine and impair mucosal repair. In the context of EMS, cAMP signaling regulates cell proliferation and apoptosis through both PKA and Epac and modulates cytoskeletal dynamics, adhesion molecules, and hydrolases, thereby influencing cell invasiveness and migration [25]. In principle, altered ADCY3 expression in ectopic endometrial cells could lead to dysregulated proliferation, increased resistance to apoptosis, and enhanced invasiveness; however, this hypothesis remains to be tested experimentally.

As a member of the heat‐shock protein family, DNAJC27 regulates protein folding and degradation through its interaction with Hsp70 and thereby helps to maintain cellular protein homeostasis. Previous work has linked DNAJC27 to activation of the MAPK signaling pathway [26]. MAPK signaling contributes to the pathogenesis of endometriosis by regulating cell proliferation, differentiation, and inflammatory responses; in IBD, its hyperactivation promotes proinflammatory cytokine and chemokine release, disrupts the intestinal epithelial barrier, and modulates T‐cell maturation and macrophage activation. Although these observations are consistent with a shared role for DNAJC27‐related signaling in both conditions, direct experimental evidence linking DNAJC27 to EMS pathogenesis is currently lacking.

At the 11q13.1 locus, CCDC88B showed the highest colocalization probability (PP.H4 = 0.938) and the CCDC88 family has been implicated in immune‐cell polarity and migration [27]. It has been identified as a susceptibility gene for IBD [28], and its dysfunction may lead to excessive or inappropriate migration of immune cells into the intestinal mucosa. Although CCDC88B has not been a primary focus of endometriosis research, its role in cell migration and invasion suggests that it could contribute to the migration and implantation of ectopic endometrial cells, a possibility that warrants further investigation.

Using three complementary approaches—positional mapping, MAGMA, and eQTL analysis—we identified eight genes with convergent evidence of pleiotropy: ADCY3, CCDC88B, GREB1, NOD2, RSPO3, SYNE1, THADA, and TRAIP. NOD2 is an established IBD susceptibility gene that maintains intestinal immune homeostasis through activation of the NF‐*κ*B signaling pathway; both loss‐ and gain‐of‐function variants have been linked to inflammatory disease [29]. It is plausible that NOD2 could promote an inflammatory microenvironment in ectopic endometrial tissue through a similar mechanism, although this has not been directly demonstrated. GREB1 is an estrogen‐responsive gene that forms a positive feedback loop with estrogen receptors; in endometriosis, estrogen‐induced GREB1 expression promotes the growth of ectopic lesions [30]. The identification of GREB1 as a pleiotropic gene suggests a potential hormonal contribution to the genetic overlap between EMS and IBD, consistent with the known influence of estrogen on intestinal inflammation. RSPO3 has been identified as a causal protein for IBD and has been implicated in the inflammation and repair mechanisms of Crohn′s disease [31]. In endometriosis, RSPO3 may contribute to tissue remodeling through the Wnt signaling pathway [32]. Collectively, the functional diversity of these pleiotropic genes suggests that the co‐occurrence of IBD and EMS may be mediated through a cross‐cutting network involving immune, hormonal, and tissue‐repair pathways. It should be emphasized that these mechanistic interpretations are based largely on existing literature and integrative bioinformatics analyses; they should be considered hypothesis‐generating rather than conclusive, and dedicated functional studies will be required for validation.

### 4.2. Pathway Enrichment and Shared Mechanisms

MAGMA gene‐set analysis identified 10 significantly enriched pathways, of which positive regulation of gene expression was the most prominent (*p* < 0.05, Bonferroni corrected; Figure 3). This pathway is consistent with the cAMP signaling function of ADCY3 and the transcriptional activation role of NOD2, suggesting that dynamic regulation of gene expression may represent a molecular basis for the co‐occurrence of the two diseases. Epigenetic modifications, such as histone acetylation, may mediate this process. Studies of DSS‐induced chronic colitis in mice have reported elevated H3K27ac levels at colonic enhancers [33], and STAT1 has been shown to recruit EP300 to enrich H3K27ac at the enhancers of inflammatory target genes such as LCP2 and TNFAIP2 in IBD [34]. In endometriosis, Monteiro et al. [35] observed hyperacetylation of histones H3 and H4 at the promoter of the SF‐1 gene in ectopic lesions, with concomitant upregulation of SF‐1 expression that promotes local estrogen production. Although these observations are suggestive of shared epigenetic mechanisms, they were derived from separate studies in different disease contexts and should therefore be interpreted with caution.

Tissue enrichment analyses revealed significant signals in whole blood, spleen, and colon, consistent with the immunoinflammatory nature of the two diseases. The enrichment in whole blood reflects systemic immune activation, and the spleen, as a lymphoid organ, may mediate immune responses through T cells and dendritic cells. Enrichment in the colon directly reflects the local pathology of IBD, whereas distal inflammation in EMS may influence the colonic microenvironment through circulating immune cells. eQTL analysis showed significant upregulation of NKX2‐3 in the colon and small intestine (Figure S6). As a transcription factor, NKX2‐3 regulates the development and homeostasis of intestinal epithelial cells, and its suppression impairs intestinal‐cell differentiation and compromises epithelial barrier integrity [36]. NKX2‐3 also regulates VEGF and EDN1 signaling in intestinal microvascular endothelial cells [36], pathways that are relevant to angiogenesis and inflammation in endometriosis [37]. Since differential gene expression in uterine tissue is hormone‐dependent, its co‐occurrence with intestinal inflammation in IBD is consistent with the hypothesis that the hormone–immune axis may contribute to the co‐occurrence of these two diseases, although direct evidence remains limited.

The PPI network (Figure S7) showed that ADCY3, NOD2, and CCDC88B formed a closely connected interaction module, suggesting that these genes may promote inflammation through signaling cascades such as the cAMP–PKA–NF‐*κ*B axis. The dynamics of this network remain to be verified by targeted proteomic studies. To our knowledge, this study is the first to combine PLACO and colocalization analysis to characterize the genetic overlap between EMS and IBD, confirming the potential role of established IBD susceptibility genes such as NOD2. Although the roles of NOD2 and ADCY3 in IBD have been extensively studied, their functions in EMS remain to be fully characterized. By contrast, hormone‐related genes characteristic of EMS, such as GREB1, appear to play a weaker role in IBD, reflecting differences between the pathogenic mechanisms of the two diseases.

### 4.3. Limitations and Future Directions

Several limitations of this study should be acknowledged. First, the datasets were derived primarily from European populations, with limited representation of other ancestral groups such as Asian and African populations, which may limit the generalizability of the findings. The genetic background of IBD differs substantially between Asian and European populations [28], and the single‐ancestry nature of the sample may increase the false‐negative rate for pleiotropic loci that are specific to other populations. Second, because of the relatively weak association signals in the endometriosis GWAS, the instrument‐selection threshold was relaxed to *p* < 5 × 10^−6^ in the reverse MR direction. Although this deviates from the conventional genome‐wide significance level of *p* < 5 × 10^−8^, such relaxation is a well‐established strategy for MR studies involving traits with few genetic instruments, as supported by methodological simulation studies [19]. To mitigate weak‐instrument bias, only SNPs with *F*‐statistics greater than 10 were retained (minimum *F* = 30.2 in the reverse analysis) and extensive sensitivity analyses were performed, including MR‐Egger, weighted median, weighted mode, DIVW, and MR‐RAPS. Third, the lack of functional experiments limits direct evidence for the causal mechanisms proposed here. The mechanistic interpretations in this study are based on integrative bioinformatics analyses and existing literature and should be regarded as hypothesis‐generating rather than definitive; future work should incorporate functional validation through in vitro and in vivo experiments. Fourth, although power analysis indicated adequate statistical power for the forward direction, the reverse direction was powered mainly to detect moderate‐to‐large effects (OR ≥ 1.20); very small causal effects of endometriosis on IBD (OR < 1.20) cannot therefore be excluded. Future studies should include multiomics integrative analyses that combine epigenomic data and spatial transcriptomics to clarify the role of the immune–hormone axis in both diseases. Transethnic GWAS analyses would also help to assess the generalizability of the pleiotropic loci identified here.

## Author Contributions

All authors contributed to the conception and design of the study. Material preparation, data collection, and analysis were performed by X.C., J.W., J.C., H.W., J.L., and Y.F., with X.C. leading the data analysis. The first draft of the manuscript was written by X.C., and all authors, including G.W., commented on previous versions. G.W. supervised the project and critically revised the manuscript.

## Funding

No funding was received for this manuscript.

## Disclosure

All authors read and approved the final manuscript.

## Ethics Statement

It is not applicable. This study used publicly available GWAS summary statistics only and did not involve human participants, individual‐level human data, or biological samples requiring ethical approval. The original GWAS studies from which the summary statistics were derived obtained appropriate ethical approvals and informed consent from participants (de Lange et al., Nature Genetics 2017; FinnGen R12).

## Consent

The authors have nothing to report.

## Conflicts of Interest

The authors declare no conflicts of interest.

## Supporting Information

Additional supporting information can be found online in the Supporting Information section.

## Supporting information


**Supporting Information 1** Figure S1: QQ plot for whole‐genome PLACO pleiotropy analysis. Figure S2: Characteristics of the pleiotropic genomic loci identified by PLACO, showing the genomic size (kb), number of candidate SNPs, number of mapped genes, and number of genes physically located within each locus. Figure S3: Functional impact of pleiotropic SNPs on genes. Figure S4: Manhattan plot of MAGMA gene analysis. Figure S5: QQ plot for MAGMA gene analysis. Figure S6: Expression of overlapping pleiotropic genes across different tissues. Figure S7: Protein–protein interaction (PPI) analysis of pleiotropic genes. Figure S8: Leave‐one‐out forest plot for forward MR (IBD → EMS). Figure S9: Leave‐one‐out forest plot for reverse MR (EMS → IBD). Figure S10: Statistical power curves for bidirectional MR at two‐sided *α* = 0.05 (mRnd framework).

**Supporting Information 2** Table S1: Shared genetic loci for IBD and endometriosis. Table S2: Gene set enrichment analysis of shared risk genes between endometriosis and IBD. Table S3: MAGMA gene‐based association results for shared loci. Table S4: Colocalization of IBD and endometriosis GWAS signals with eQTLs. Table S5: Functional annotation of nearby genes at shared loci. Table S6: Tissue‐wide expression profiles of candidate shared genes (GTEx). Table S7: GO and KEGG pathway enrichment of shared genes.

**Supporting Information 3** Table S8: Forward MR instrumental variables (109 SNPs): rsID, chromosome, position, effect/other allele, exposure beta/SE/P, outcome beta/SE/P, *F*‐statistic. Table S9: Reverse MR instrumental variables (21 SNPs): same columns as Table S8. Table S10: Leave‐one‐out sensitivity analysis: IVW estimates after excluding each SNP in turn (109 forward + 21 reverse rows, plus all‐SNPs rows). Table S11: Statistical power at multiple detectable odds ratios, computed with the mRnd framework.

## Data Availability

The GWAS summary statistics used in this study are publicly available from the following sources: (1) endometriosis data were obtained from the FinnGen R12 release (https://r12.finngen.fi/), including 20,190 cases and 130,160 controls and (2) IBD data were obtained from a published GWAS meta‐analysis (de Lange et al., Nature Genetics 2017, PMID: 28067908) that included 25,305 participants and identified 25 novel susceptibility loci. All data are summary‐level statistics from publicly available resources and did not require individual‐level access or additional ethical approval. The complete list of pleiotropic SNPs identified by PLACO is provided in Table S1. The complete per‐SNP instrumental‐variable data used in the Mendelian randomization analyses, including rsIDs, positions, alleles, effect sizes, standard errors, *p* values, and *F*‐statistics, are provided in Tables S8 (109 forward instruments) and S9 (21 reverse instruments). Per‐SNP leave‐one‐out results and statistical‐power calculations at multiple detectable odds ratios are reported in Tables S10 and S11. Complete analysis scripts and parameter files are provided as a reproducibility package accompanying this submission; additional materials are available from the corresponding author on reasonable request.
