# Supplementary material for: Shared Genetic Architecture and Pleiotropic Loci Linking Endometriosis and Inflammatory Bowel Disease: An Integrative GWAS, Colocalization, and Mendelian Randomization Study
Source: Biomed Res Int. 2026 Jul 27;2026:8422273. doi: 10.1155/bmri/8422273 (PMC13408434; doi:10.1155/bmri/8422273)
Supplement: Supplementary file 1 — Supporting Information 1 Figure S1: QQ plot for whole‐genome PLACO pleiotropy analysis. Figure S2: Characteristics of the pleiotropic genomic loci identified by PLACO, showing the genomic size (kb), number of candidate SNPs, number of mapped genes, and number of genes physically located within each locus. Figure S3: Functional impact of pleiotropic SNPs on genes. Figure S4: Manhattan plot of MAGMA gene analysis. Figure S5: QQ plot for MAGMA gene analysis. Figure S6: Expression of overlapping pleiotropic genes across different tissues. Figure S7: Protein–protein interaction (PPI) analysis of pleiotropic genes. Figure S8: Leave‐one‐out forest plot for forward MR (IBD → EMS). Figure S9: Leave‐one‐out forest plot for reverse MR (EMS → IBD). Figure S10: Statistical power curves for bidirectional MR at two‐sided α = 0.05 (mRnd framework). [file BMRI-2026-8422273-s001.docx]

## Supplementary Figures


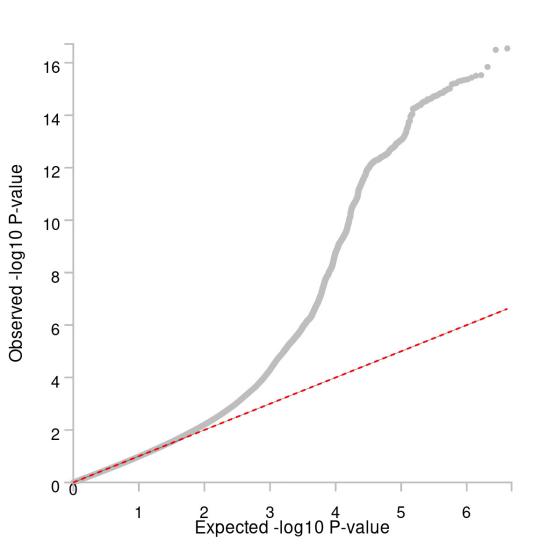


Figure S1 QQ plot for whole-genome PLACO pleiotropy analysis


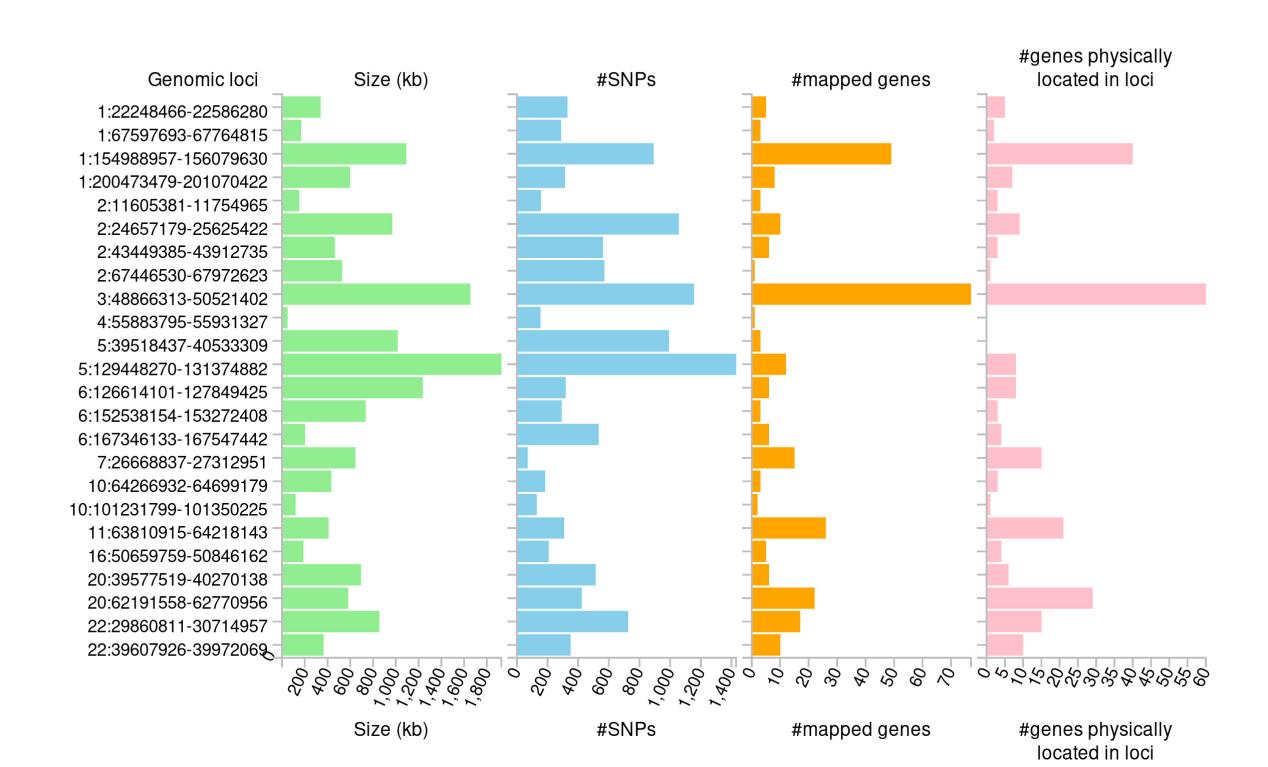


Figure S2. Characteristics of the pleiotropic genomic loci identified by PLACO. For each locus (labelled by its chromosomal boundaries), the panels show the genomic size (kb), the number of candidate SNPs, the number of mapped genes, and the number of genes physically located within the locus.


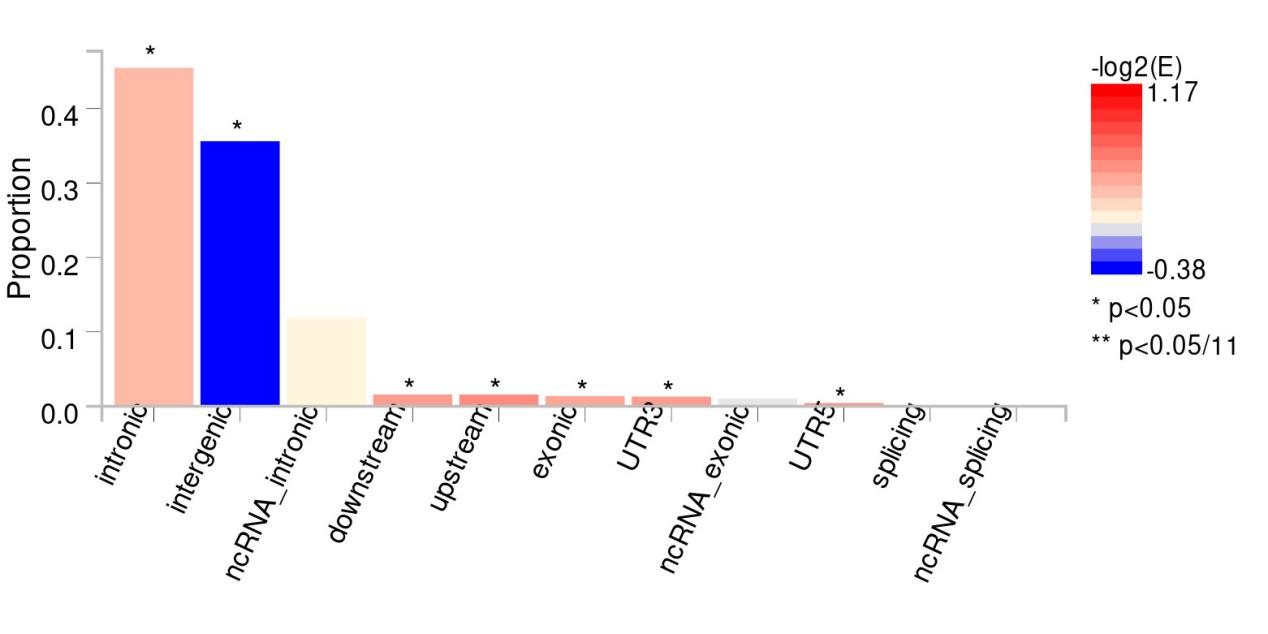


Figure S3 Functional impact of a pleiotropic SNP on genes


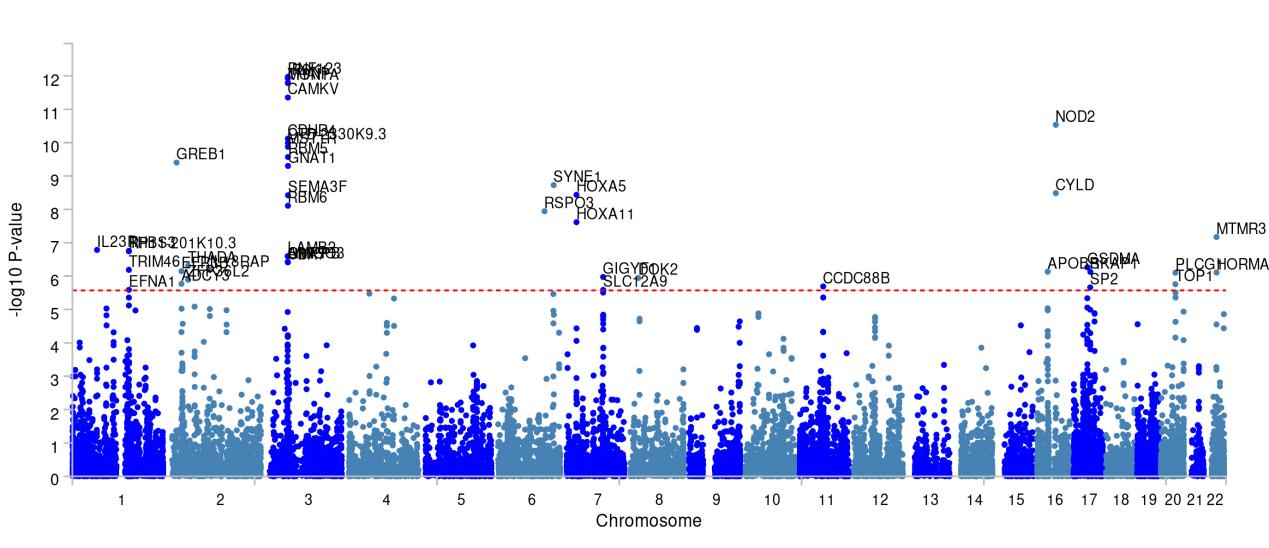


Figure S4 Manhattan plot of MAGMA gene analysis


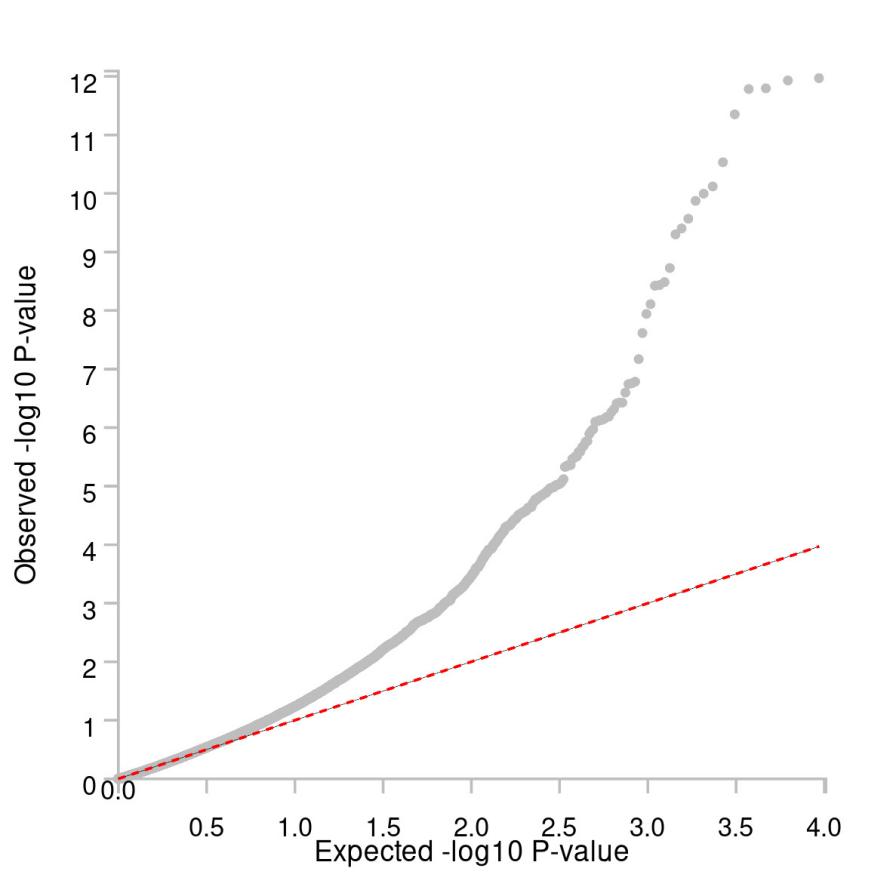


Figure S5 QQ plot from MAGMA gene analysis


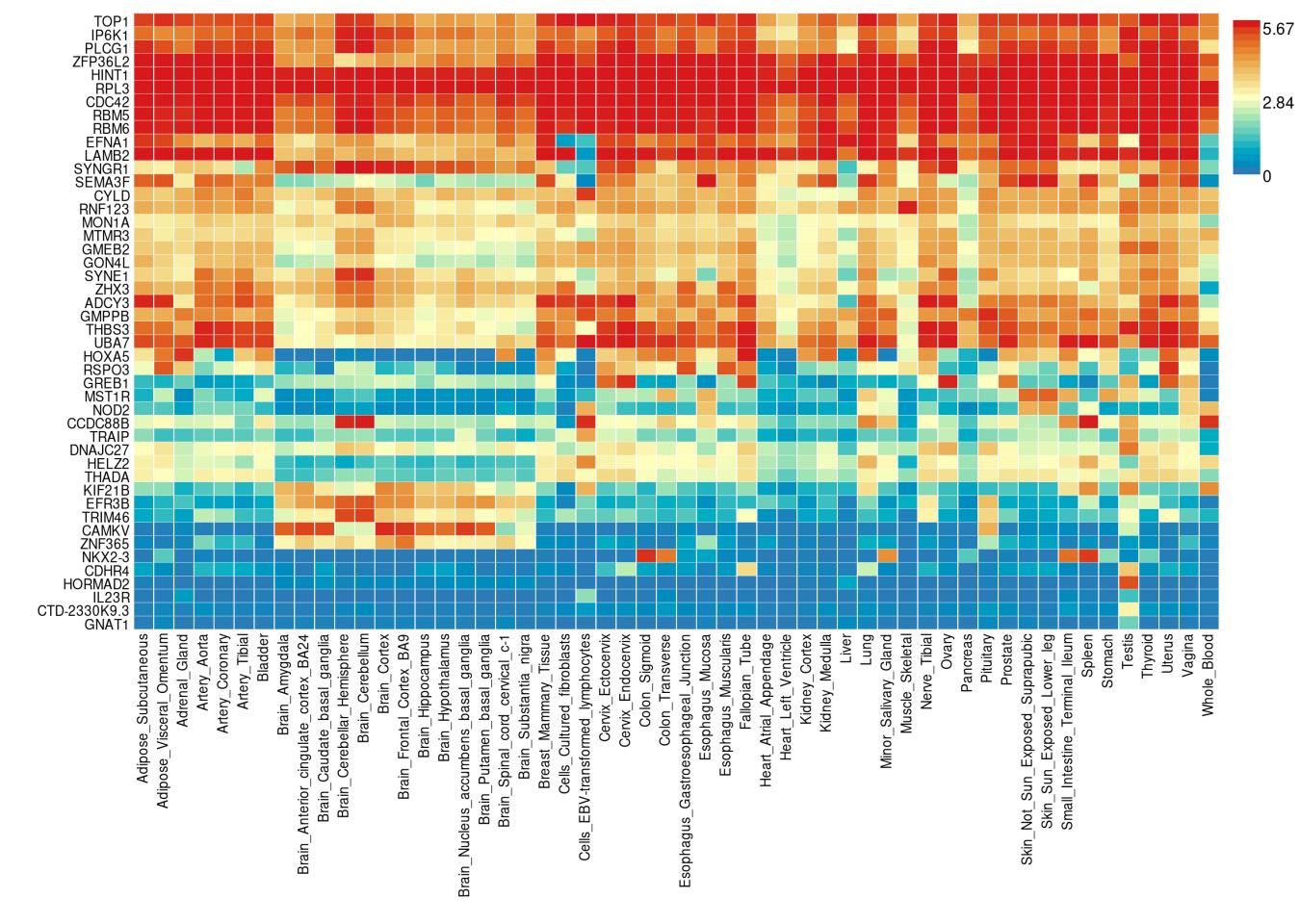


Figure S6. Expression of overlapping pleiotropic genes across different tissues

**
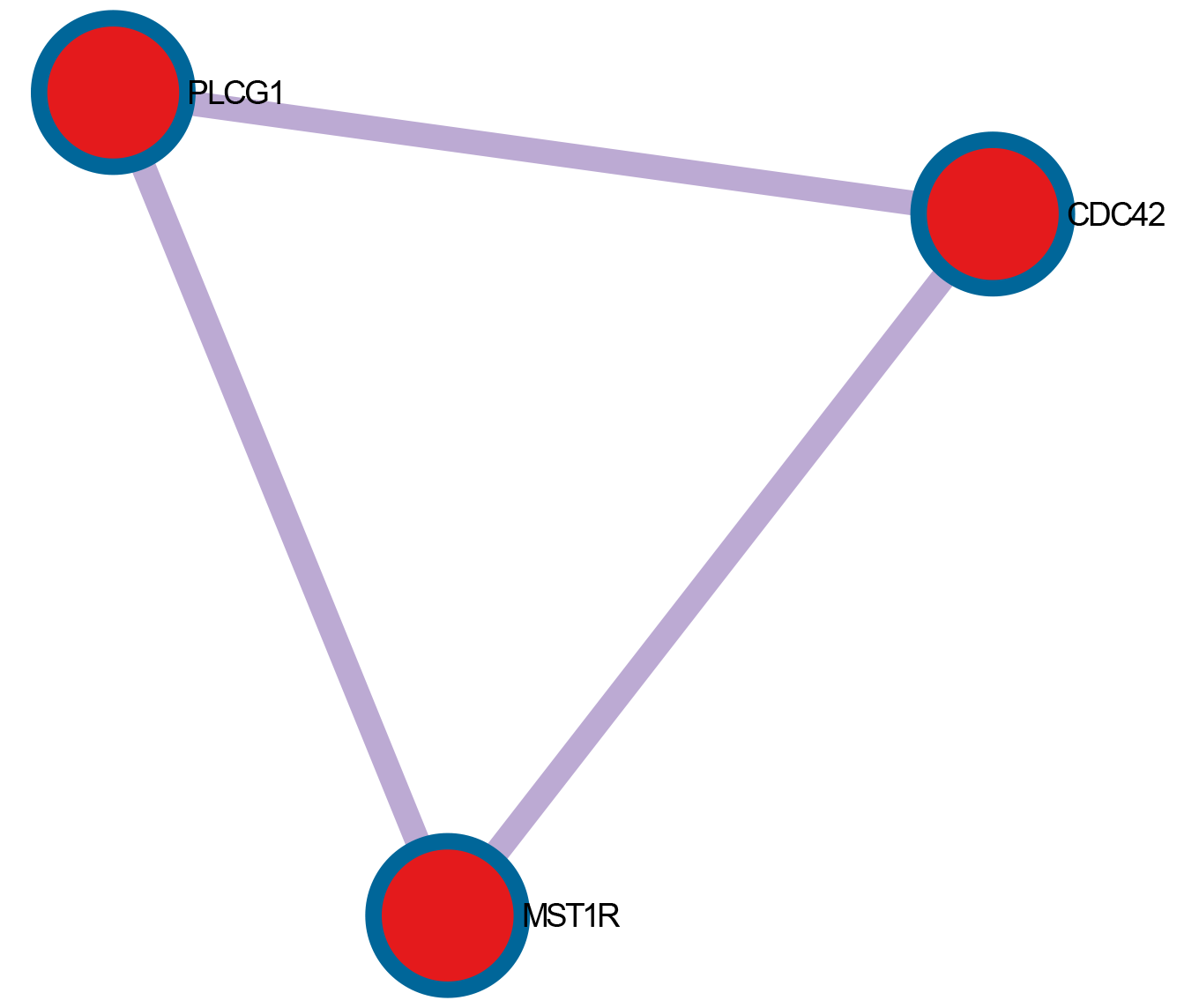
**

Figure S7. Analysis of Protein-Protein Interactions(PPI) for pleiotropic genes

**
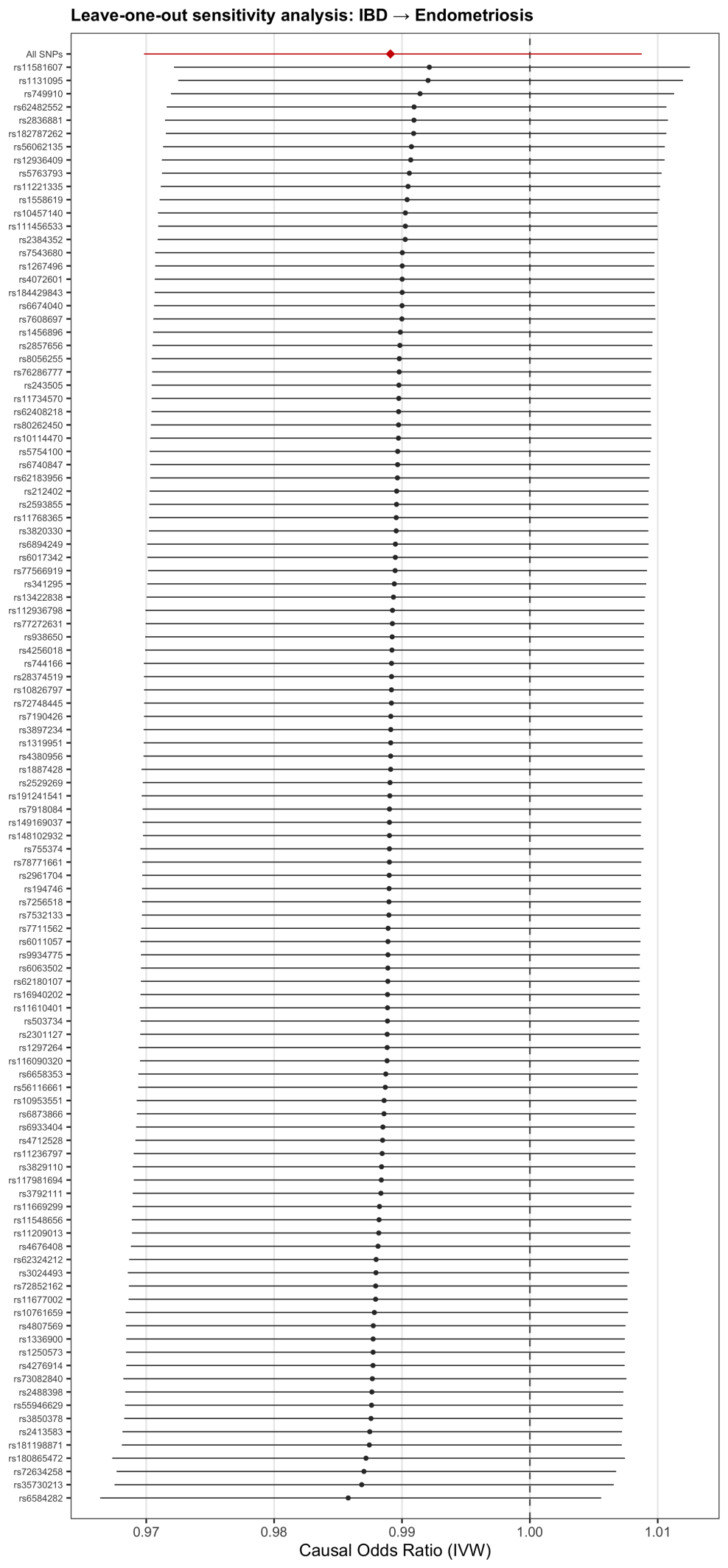
**

Figure S8. Leave-one-out forest plot for forward MR (IBD → EMS)


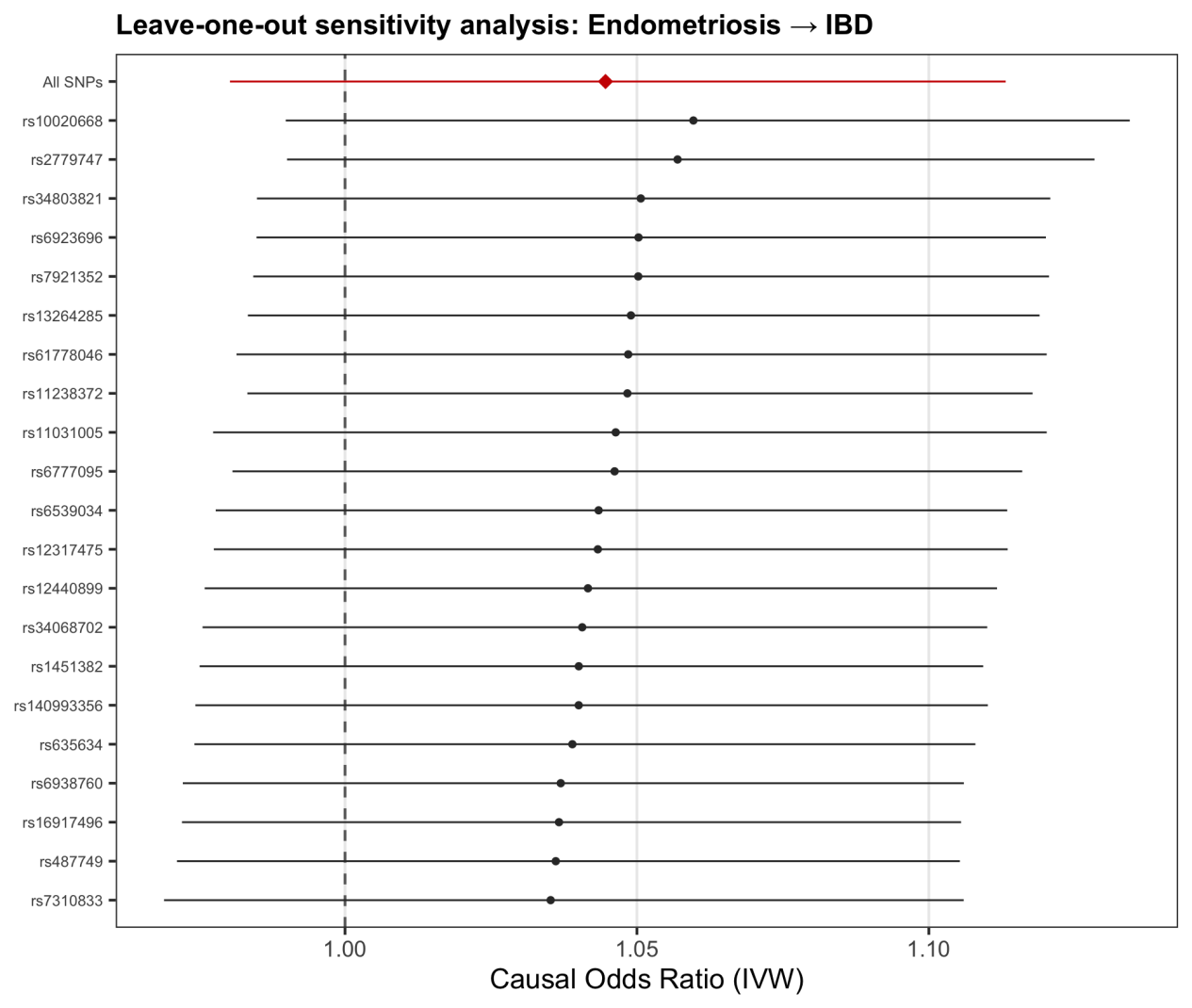


Figure S9. Leave-one-out forest plot for reverse MR (EMS → IBD)


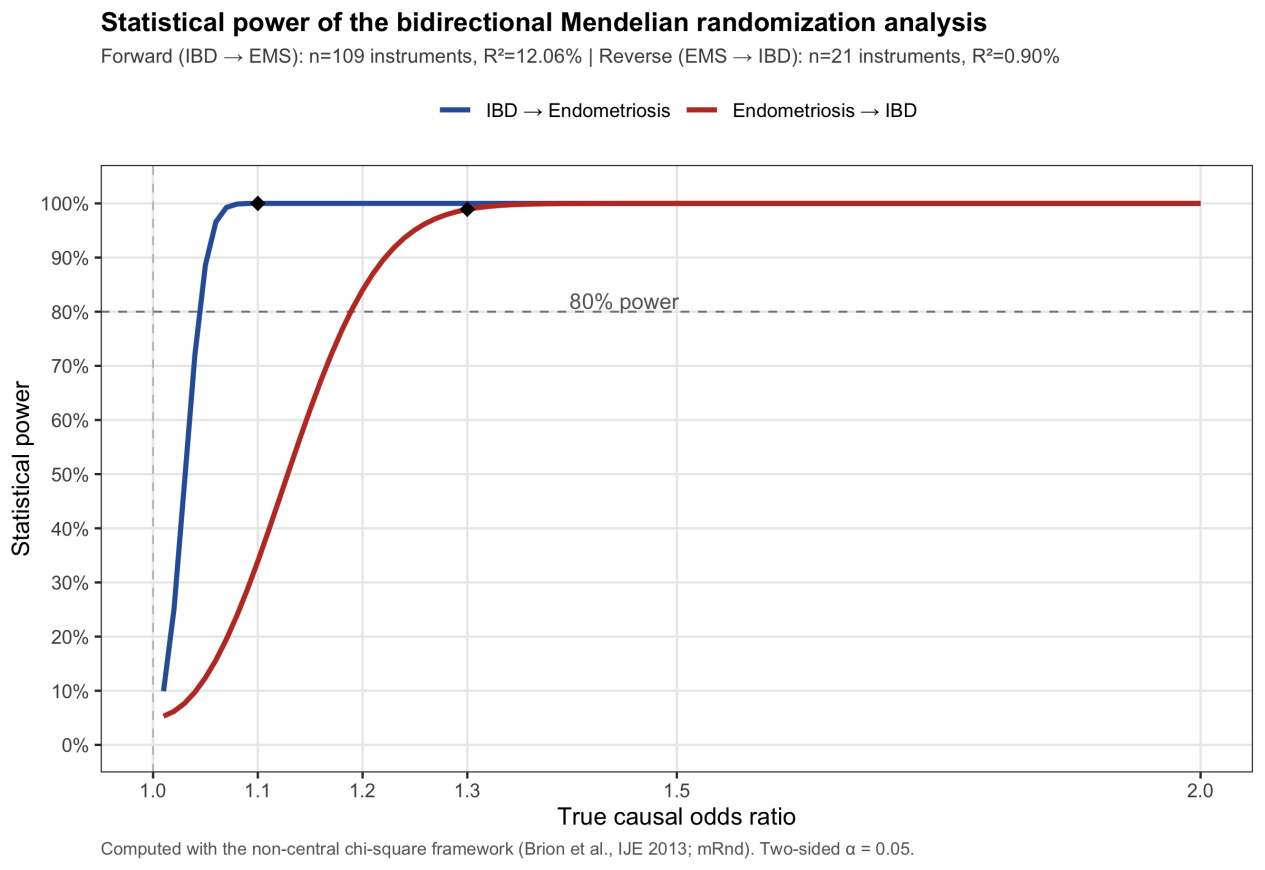


Figure S10. Statistical power curves for bidirectional MR at two-sided α = 0.05 (mRnd framework)
